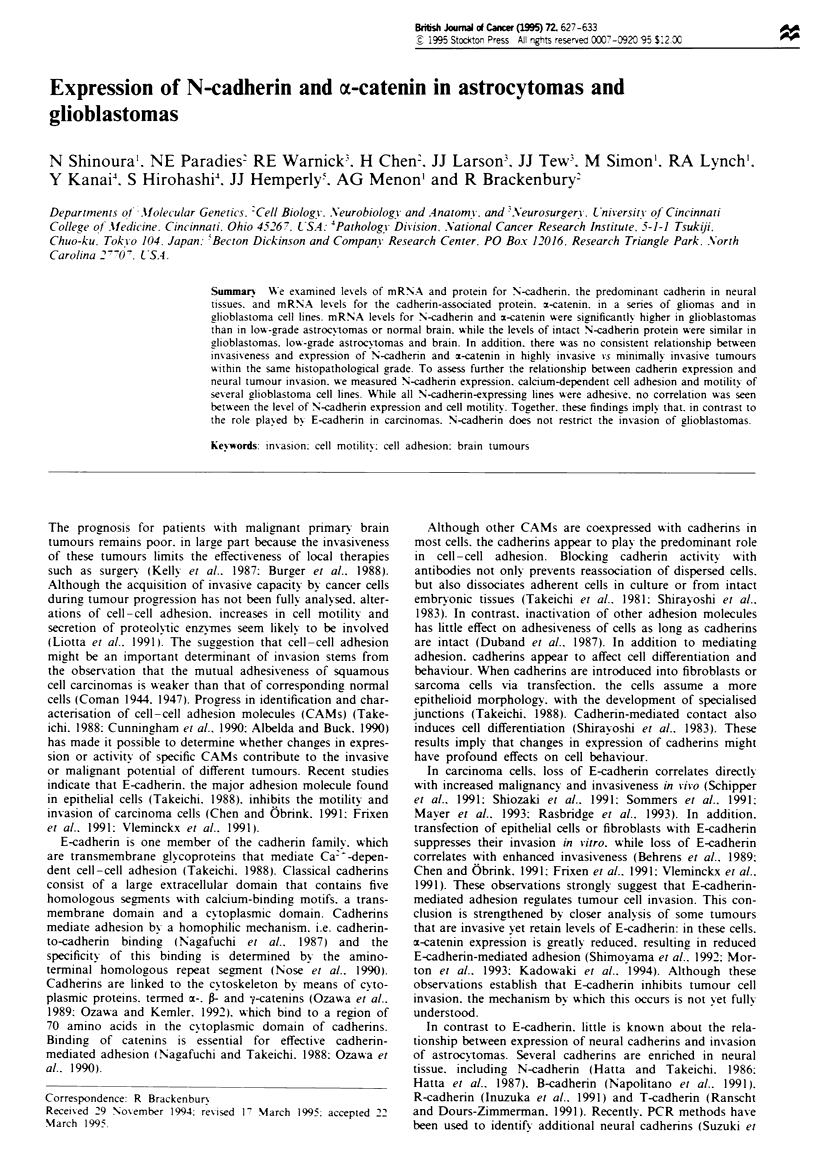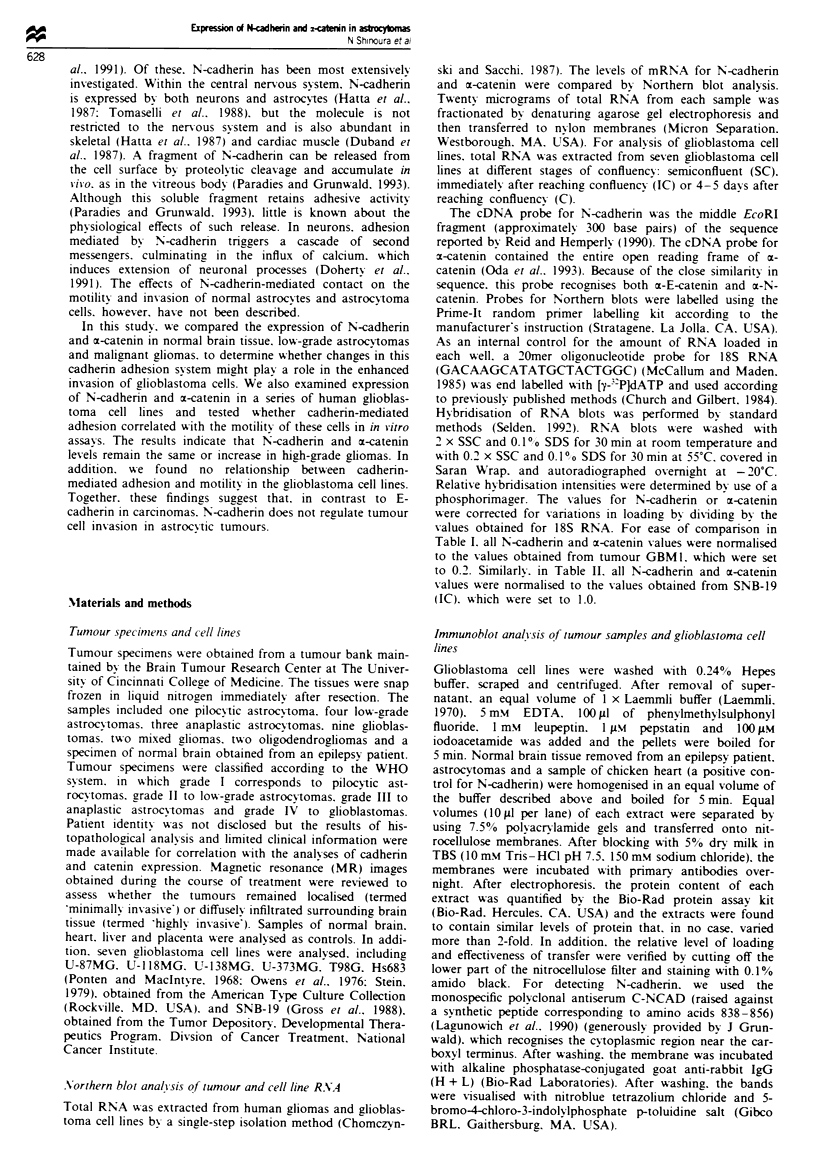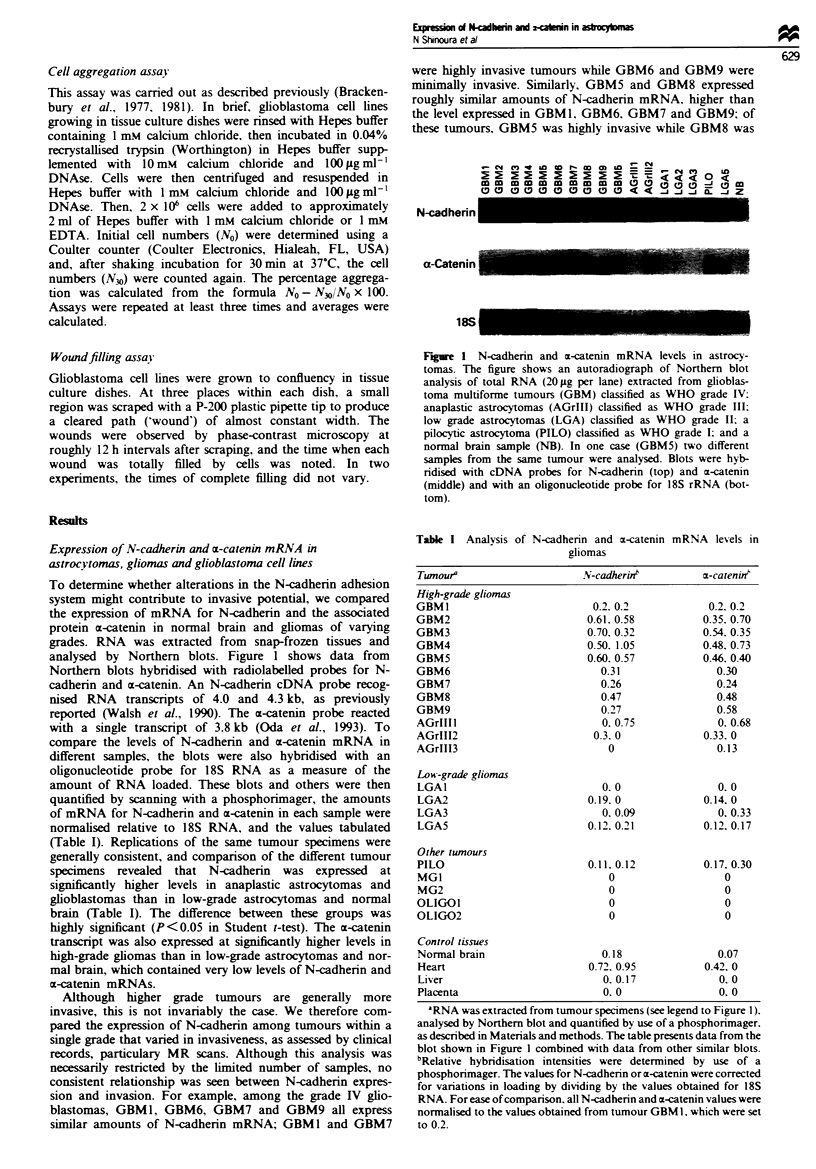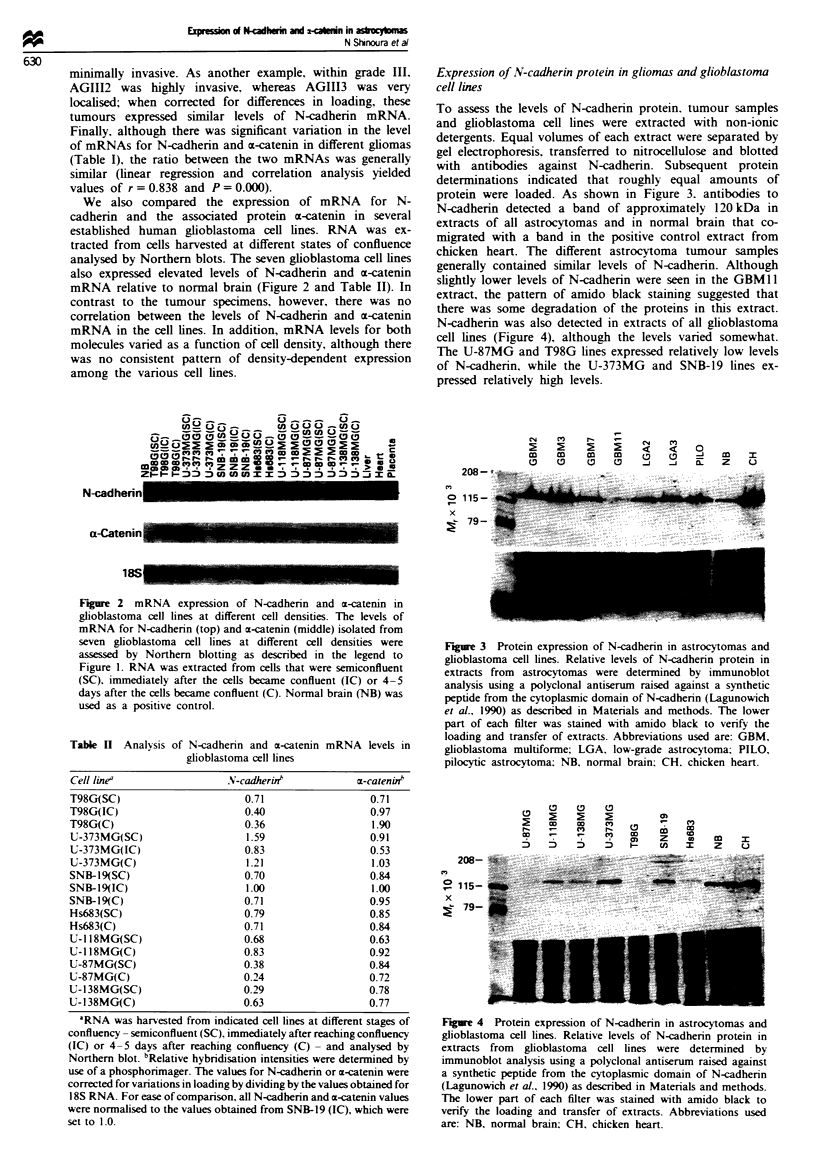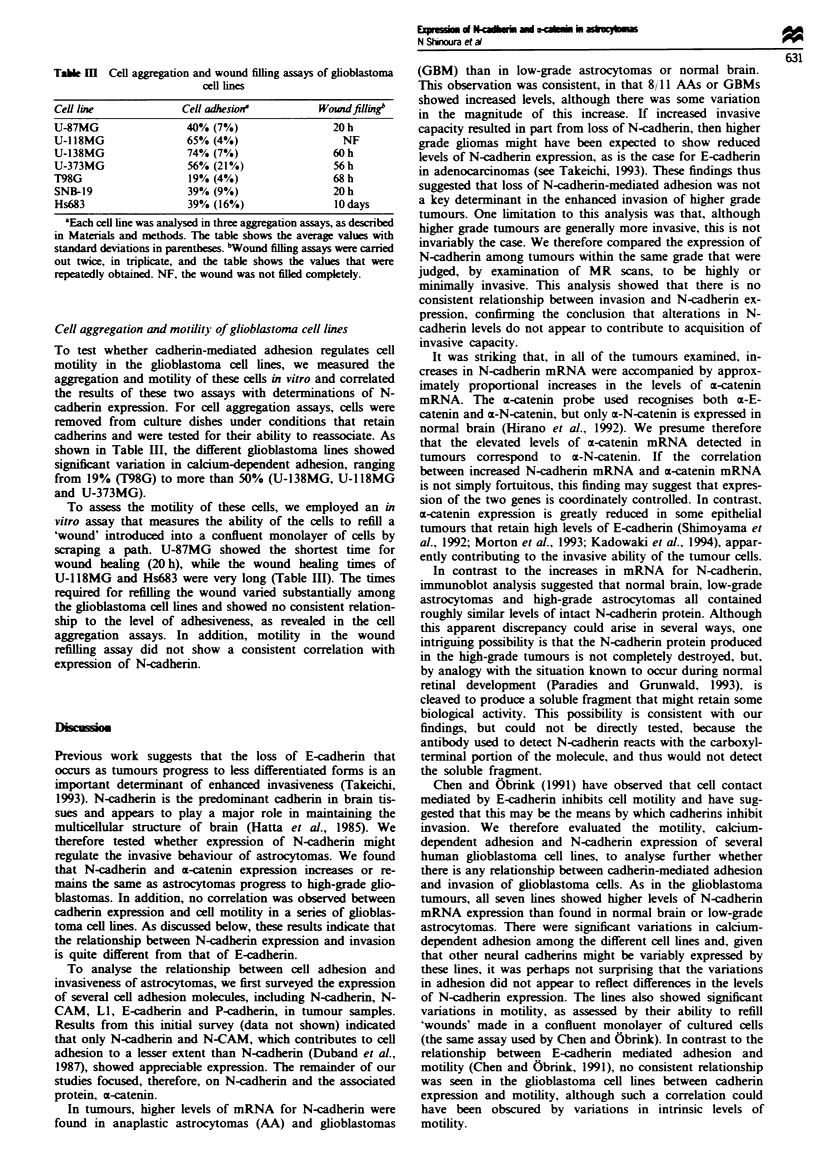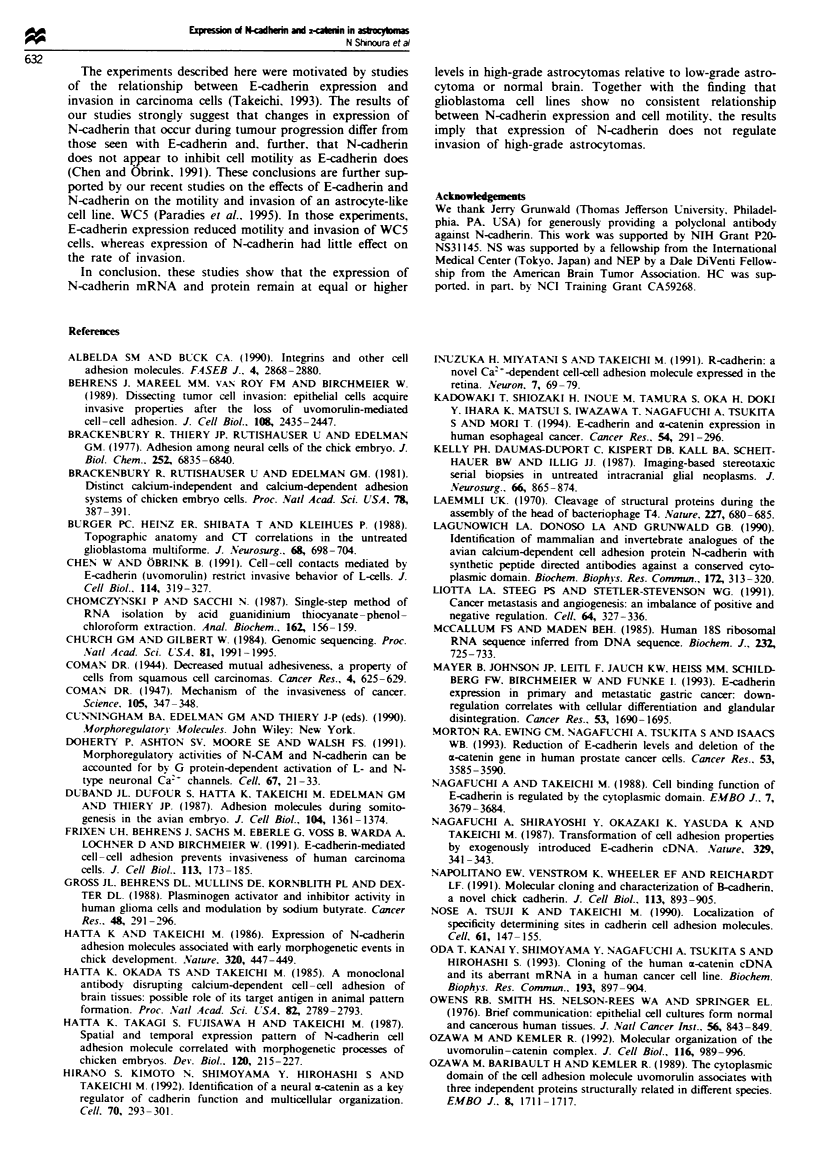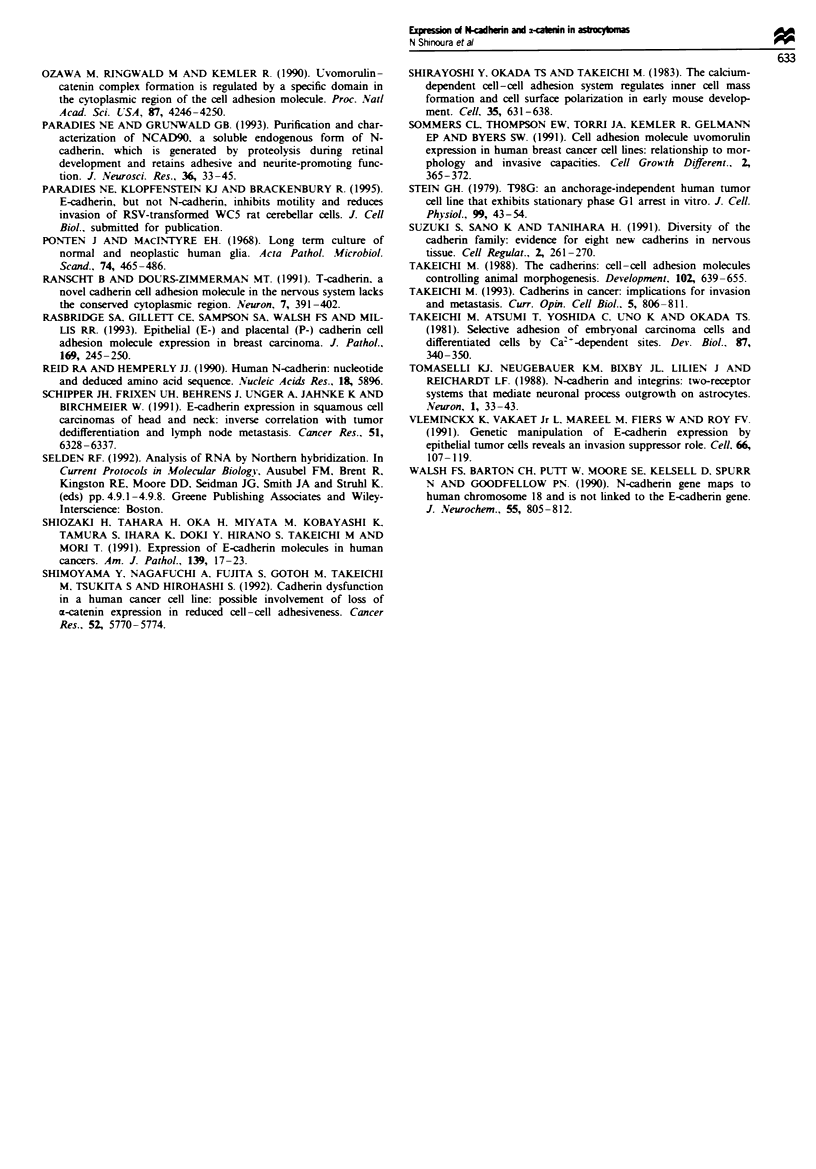# Expression of N-cadherin and alpha-catenin in astrocytomas and glioblastomas.

**DOI:** 10.1038/bjc.1995.384

**Published:** 1995-09

**Authors:** N. Shinoura, N. E. Paradies, R. E. Warnick, H. Chen, J. J. Larson, J. J. Tew, M. Simon, R. A. Lynch, Y. Kanai, S. Hirohashi

**Affiliations:** Department of Molecular Genetics, University of Cincinnati, College of Medicine, Ohio 45267, USA.

## Abstract

**Images:**


British Journal of Cancer (1995) 72, 627-633

© 1995 Stockton Press All rights reserved 0007-0920/95 $12.00

### Expression of N-cadherin and α-catenin in astrocytomas and glioblastomas

N Shinoura[1], NE Paradies[2] RE Warnick[3], H Chen[2], JJ Larson[3], JJ Tew[3], M Simon[1], RA Lynch[1], Y Kanai[4], S Hirohashi[4], JJ Hemperly[5], AG Menon[1] and R Brackenbury[2]

*Departments of [1]Molecular Genetics, [2]Cell Biology, Neurobiology and Anatomy, and [3]Neurosurgery, University of Cincinnati College of Medicine, Cincinnati, Ohio 45267, USA; [4]Pathology Division, National Cancer Research Institute, 5-1-1 Tsukiji, Chuo-ku, Tokyo 104, Japan; [5]Becton Dickinson and Company Research Center, PO Box 12016, Research Triangle Park, North Carolina 27707, USA.*

**Summary** We examined levels of mRNA and protein for N-cadherin, the predominant cadherin in neural tissues, and mRNA levels for the cadherin-associated protein, α-catenin, in a series of gliomas and in glioblastoma cell lines. mRNA levels for N-cadherin and α-catenin were significantly higher in glioblastomas than in low-grade astrocytomas or normal brain, while the levels of intact N-cadherin protein were similar in glioblastomas, low-grade astrocytomas and brain. In addition, there was no consistent relationship between invasiveness and expression of N-cadherin and α-catenin in highly invasive *vs* minimally invasive tumours within the same histopathological grade. To assess further the relationship between cadherin expression and neural tumour invasion, we measured N-cadherin expression, calcium-dependent cell adhesion and motility of several glioblastoma cell lines. While all N-cadherin-expressing lines were adhesive, no correlation was seen between the level of N-cadherin expression and cell motility. Together, these findings imply that, in contrast to the role played by E-cadherin in carcinomas, N-cadherin does not restrict the invasion of glioblastomas.

**Keywords**: invasion; cell motility; cell adhesion; brain tumours

The prognosis for patients with malignant primary brain tumours remains poor, in large part because the invasiveness of these tumours limits the effectiveness of local therapies such as surgery (Kelly *et al*., 1987; Burger *et al*., 1988). Although the acquisition of invasive capacity by cancer cells during tumour progression has not been fully analysed, alterations of cell-cell adhesion, increases in cell motility and secretion of proteolytic enzymes seem likely to be involved (Liotta *et al*., 1991). The suggestion that cell-cell adhesion might be an important determinant of invasion stems from the observation that the mutual adhesiveness of squamous cell carcinomas is weaker than that of corresponding normal cells (Coman 1944, 1947). Progress in identification and characterisation of cell-cell adhesion molecules (CAMs) (Takeichi, 1988; Cunningham *et al*., 1990; Albelda and Buck, 1990) has made it possible to determine whether changes in expression or activity of specific CAMs contribute to the invasive or malignant potential of different tumours. Recent studies indicate that E-cadherin, the major adhesion molecule found in epithelial cells (Takeichi, 1988), inhibits the motility and invasion of carcinoma cells (Chen and Öbrink, 1991; Frixen *et al*., 1991; Vleminckx *et al*., 1991).

E-cadherin is one member of the cadherin family, which are transmembrane glycoproteins that mediate $Ca^{2+}$-dependent cell-cell adhesion (Takeichi, 1988). Classical cadherins consist of a large extracellular domain that contains five homologous segments with calcium-binding motifs, a transmembrane domain and a cytoplasmic domain. Cadherins mediate adhesion by a homophilic mechanism, i.e. cadherin-to-cadherin binding (Nagafuchi *et al*., 1987) and the specificity of this binding is determined by the amino-terminal homologous repeat segment (Nose *et al*., 1990). Cadherins are linked to the cytoskeleton by means of cytoplasmic proteins, termed α-, β- and γ-catenins (Ozawa *et al*., 1989; Ozawa and Kemler, 1992), which bind to a region of 70 amino acids in the cytoplasmic domain of cadherins. Binding of catenins is essential for effective cadherin-mediated adhesion (Nagafuchi and Takeichi, 1988; Ozawa *et al*., 1990).

Correspondence: R Brackenbury
Received 29 November 1994; revised 17 March 1995; accepted 22 March 1995.

Although other CAMs are coexpressed with cadherins in most cells, the cadherins appear to play the predominant role in cell-cell adhesion. Blocking cadherin activity with antibodies not only prevents reassociation of dispersed cells, but also dissociates adherent cells in culture or from intact embryonic tissues (Takeichi *et al*., 1981; Shirayoshi *et al*., 1983). In contrast, inactivation of other adhesion molecules has little effect on adhesiveness of cells as long as cadherins are intact (Duband *et al*., 1987). In addition to mediating adhesion, cadherins appear to affect cell differentiation and behaviour. When cadherins are introduced into fibroblasts or sarcoma cells via transfection, the cells assume a more epithelioid morphology, with the development of specialised junctions (Takeichi, 1988). Cadherin-mediated contact also induces cell differentiation (Shirayoshi *et al*., 1983). These results imply that changes in expression of cadherins might have profound effects on cell behaviour.

In carcinoma cells, loss of E-cadherin correlates directly with increased malignancy and invasiveness *in vivo* (Schipper *et al*., 1991; Shiozaki *et al*., 1991; Sommers *et al*., 1991; Mayer *et al*., 1993; Rasbridge *et al*., 1993). In addition, transfection of epithelial cells or fibroblasts with E-cadherin suppresses their invasion *in vitro*, while loss of E-cadherin correlates with enhanced invasiveness (Behrens *et al*., 1989; Chen and Öbrink, 1991; Frixen *et al*., 1991; Vleminckx *et al*., 1991). These observations strongly suggest that E-cadherin-mediated adhesion regulates tumour cell invasion. This conclusion is strengthened by closer analysis of some tumours that are invasive yet retain levels of E-cadherin: in these cells, α-catenin expression is greatly reduced, resulting in reduced E-cadherin-mediated adhesion (Shimoyama *et al*., 1992; Morton *et al*., 1993; Kadowaki *et al*., 1994). Although these observations establish that E-cadherin inhibits tumour cell invasion, the mechanism by which this occurs is not yet fully understood.

In contrast to E-cadherin, little is known about the relationship between expression of neural cadherins and invasion of astrocytomas. Several cadherins are enriched in neural tissue, including N-cadherin (Hatta and Takeichi, 1986; Hatta *et al*., 1987), B-cadherin (Napolitano *et al*., 1991), R-cadherin (Inuzuka *et al*., 1991) and T-cadherin (Ranscht and Dours-Zimmerman, 1991). Recently, PCR methods have been used to identify additional neural cadherins (Suzuki *et*

al., 1991). Of these, N-cadherin has been most extensively investigated. Within the central nervous system, N-cadherin is expressed by both neurons and astrocytes (Hatta et al., 1987; Tomaselli et al., 1988), but the molecule is not restricted to the nervous system and is also abundant in skeletal (Hatta et al., 1987) and cardiac muscle (Duband et al., 1987). A fragment of N-cadherin can be released from the cell surface by proteolytic cleavage and accumulate *in vivo*, as in the vitreous body (Paradies and Grunwald, 1993). Although this soluble fragment retains adhesive activity (Paradies and Grunwald, 1993), little is known about the physiological effects of such release. In neurons, adhesion mediated by N-cadherin triggers a cascade of second messengers, culminating in the influx of calcium, which induces extension of neuronal processes (Doherty et al., 1991). The effects of N-cadherin-mediated contact on the motility and invasion of normal astrocytes and astrocytoma cells, however, have not been described.

In this study, we compared the expression of N-cadherin and α-catenin in normal brain tissue, low-grade astrocytomas and malignant gliomas, to determine whether changes in this cadherin adhesion system might play a role in the enhanced invasion of glioblastoma cells. We also examined expression of N-cadherin and α-catenin in a series of human glioblastoma cell lines and tested whether cadherin-mediated adhesion correlated with the motility of these cells in *in vitro* assays. The results indicate that N-cadherin and α-catenin levels remain the same or increase in high-grade gliomas. In addition, we found no relationship between cadherin-mediated adhesion and motility in the glioblastoma cell lines. Together, these findings suggest that, in contrast to E-cadherin in carcinomas, N-cadherin does not regulate tumour cell invasion in astrocytic tumours.

### Materials and methods

### *Tumour specimens and cell lines*

Tumour specimens were obtained from a tumour bank maintained by the Brain Tumour Research Center at The University of Cincinnati College of Medicine. The tissues were snap frozen in liquid nitrogen immediately after resection. The samples included one pilocytic astrocytoma, four low-grade astrocytomas, three anaplastic astrocytomas, nine glioblastomas, two mixed gliomas, two oligodendrogliomas and a specimen of normal brain obtained from an epilepsy patient. Tumour specimens were classified according to the WHO system, in which grade I corresponds to pilocytic astrocytomas, grade II to low-grade astrocytomas, grade III to anaplastic astrocytomas and grade IV to glioblastomas. Patient identity was not disclosed but the results of histopathological analysis and limited clinical information were made available for correlation with the analyses of cadherin and catenin expression. Magnetic resonance (MR) images obtained during the course of treatment were reviewed to assess whether the tumours remained localised (termed 'minimally invasive') or diffusely infiltrated surrounding brain tissue (termed 'highly invasive'). Samples of normal brain, heart, liver and placenta were analysed as controls. In addition, seven glioblastoma cell lines were analysed, including U-87MG, U-118MG, U-138MG, U-373MG, T98G, Hs683 (Ponten and MacIntyre, 1968; Owens et al., 1976; Stein, 1979), obtained from the American Type Culture Collection (Rockville, MD, USA), and SNB-19 (Gross et al., 1988), obtained from the Tumor Depository, Developmental Therapeutics Program, Divsion of Cancer Treatment, National Cancer Institute.

### *Northern blot analysis of tumour and cell line RNA*

Total RNA was extracted from human gliomas and glioblastoma cell lines by a single-step isolation method (Chomczynski and Sacchi, 1987). The levels of mRNA for N-cadherin and α-catenin were compared by Northern blot analysis. Twenty micrograms of total RNA from each sample was fractionated by denaturing agarose gel electrophoresis and then transferred to nylon membranes (Micron Separation, Westborough, MA, USA). For analysis of glioblastoma cell lines, total RNA was extracted from seven glioblastoma cell lines at different stages of confluency: semiconfluent (SC), immediately after reaching confluency (IC) or 4–5 days after reaching confluency (C).

The cDNA probe for N-cadherin was the middle *Eco*RI fragment (approximately 300 base pairs) of the sequence reported by Reid and Hemperly (1990). The cDNA probe for α-catenin contained the entire open reading frame of α-catenin (Oda et al., 1993). Because of the close similarity in sequence, this probe recognises both α-E-catenin and α-N-catenin. Probes for Northern blots were labelled using the Prime-It random primer labelling kit according to the manufacturer's instruction (Stratagene, La Jolla, CA, USA). As an internal control for the amount of RNA loaded in each well, a 20mer oligonucleotide probe for 18S RNA (GACAAGCATATGCTACTGGC) (McCallum and Maden, 1985) was end labelled with [$\gamma$-$^{32}$P]dATP and used according to previously published methods (Church and Gilbert, 1984). Hybridisation of RNA blots was performed by standard methods (Selden, 1992). RNA blots were washed with 2 × SSC and 0.1% SDS for 30 min at room temperature and with 0.2 × SSC and 0.1% SDS for 30 min at 55°C, covered in Saran Wrap, and autoradiographed overnight at −20°C. Relative hybridisation intensities were determined by use of a phosphorimager. The values for N-cadherin or α-catenin were corrected for variations in loading by dividing by the values obtained for 18S RNA. For ease of comparison in Table I, all N-cadherin and α-catenin values were normalised to the values obtained from tumour GBM1, which were set to 0.2. Similarly, in Table II, all N-cadherin and α-catenin values were normalised to the values obtained from SNB-19 (IC), which were set to 1.0.

### *Immunoblot analysis of tumour samples and glioblastoma cell lines*

Glioblastoma cell lines were washed with 0.24% Hepes buffer, scraped and centrifuged. After removal of supernatant, an equal volume of 1 × Laemmli buffer (Laemmli, 1970), 5 mM EDTA, 100 µl of phenylmethylsulphonyl fluoride, 1 mM leupeptin, 1 µM pepstatin and 100 µM iodoacetamide was added and the pellets were boiled for 5 min. Normal brain tissue removed from an epilepsy patient, astrocytomas and a sample of chicken heart (a positive control for N-cadherin) were homogenised in an equal volume of the buffer described above and boiled for 5 min. Equal volumes (10 µl per lane) of each extract were separated by using 7.5% polyacrylamide gels and transferred onto nitrocellulose membranes. After blocking with 5% dry milk in TBS (10 mM Tris–HCl pH 7.5, 150 mM sodium chloride), the membranes were incubated with primary antibodies overnight. After electrophoresis, the protein content of each extract was quantified by the Bio-Rad protein assay kit (Bio-Rad, Hercules, CA, USA) and the extracts were found to contain similar levels of protein that, in no case, varied more than 2-fold. In addition, the relative level of loading and effectiveness of transfer were verified by cutting off the lower part of the nitrocellulose filter and staining with 0.1% amido black. For detecting N-cadherin, we used the monospecific polyclonal antiserum C-NCAD (raised against a synthetic peptide corresponding to amino acids 838–856) (Lagunowich et al., 1990) (generously provided by J Grunwald), which recognises the cytoplasmic region near the carboxyl terminus. After washing, the membrane was incubated with alkaline phosphatase-conjugated goat anti-rabbit IgG (H + L) (Bio-Rad Laboratories). After washing, the bands were visualised with nitroblue tetrazolium chloride and 5-bromo-4-chloro-3-indolylphosphate p-toluidine salt (Gibco BRL, Gaithersburg, MA, USA).

*Cell aggregation assay*

This assay was carried out as described previously (Brackenbury *et al.*, 1977, 1981). In brief, glioblastoma cell lines growing in tissue culture dishes were rinsed with Hepes buffer containing 1 mM calcium chloride, then incubated in 0.04% recrystallised trypsin (Worthington) in Hepes buffer supplemented with 10 mM calcium chloride and 100 $\mu g\ ml^{-1}$ DNAse. Cells were then centrifuged and resuspended in Hepes buffer with 1 mM calcium chloride and 100 $\mu g\ ml^{-1}$ DNAse. Then, $2 \times 10^6$ cells were added to approximately 2 ml of Hepes buffer with 1 mM calcium chloride or 1 mM EDTA. Initial cell numbers ($N_0$) were determined using a Coulter counter (Coulter Electronics, Hialeah, FL, USA) and, after shaking incubation for 30 min at 37°C, the cell numbers ($N_{30}$) were counted again. The percentage aggregation was calculated from the formula $N_0 - N_{30}/N_0 \times 100$. Assays were repeated at least three times and averages were calculated.

*Wound filling assay*

Glioblastoma cell lines were grown to confluency in tissue culture dishes. At three places within each dish, a small region was scraped with a P-200 plastic pipette tip to produce a cleared path ('wound') of almost constant width. The wounds were observed by phase-contrast microscopy at roughly 12 h intervals after scraping, and the time when each wound was totally filled by cells was noted. In two experiments, the times of complete filling did not vary.

### Results

*Expression of N-cadherin and α-catenin mRNA in astrocytomas, gliomas and glioblastoma cell lines*

To determine whether alterations in the N-cadherin adhesion system might contribute to invasive potential, we compared the expression of mRNA for N-cadherin and the associated protein α-catenin in normal brain and gliomas of varying grades. RNA was extracted from snap-frozen tissues and analysed by Northern blots. Figure 1 shows data from Northern blots hybridised with radiolabelled probes for N-cadherin and α-catenin. An N-cadherin cDNA probe recognised RNA transcripts of 4.0 and 4.3 kb, as previously reported (Walsh *et al.*, 1990). The α-catenin probe reacted with a single transcript of 3.8 kb (Oda *et al.*, 1993). To compare the levels of N-cadherin and α-catenin mRNA in different samples, the blots were also hybridised with an oligonucleotide probe for 18S RNA as a measure of the amount of RNA loaded. These blots and others were then quantified by scanning with a phosphorimager, the amounts of mRNA for N-cadherin and α-catenin in each sample were normalised relative to 18S RNA, and the values tabulated (Table I). Replications of the same tumour specimens were generally consistent, and comparison of the different tumour specimens revealed that N-cadherin was expressed at significantly higher levels in anaplastic astrocytomas and glioblastomas than in low-grade astrocytomas and normal brain (Table I). The difference between these groups was highly significant ($P < 0.05$ in Student *t*-test). The α-catenin transcript was also expressed at significantly higher levels in high-grade gliomas than in low-grade astrocytomas and normal brain, which contained very low levels of N-cadherin and α-catenin mRNAs.

Although higher grade tumours are generally more invasive, this is not invariably the case. We therefore compared the expression of N-cadherin among tumours within a single grade that varied in invasiveness, as assessed by clinical records, particulary MR scans. Although this analysis was necessarily restricted by the limited number of samples, no consistent relationship was seen between N-cadherin expression and invasion. For example, among the grade IV glioblastomas, GBM1, GBM6, GBM7 and GBM9 all express similar amounts of N-cadherin mRNA; GBM1 and GBM7 were highly invasive tumours while GBM6 and GBM9 were minimally invasive. Similarly, GBM5 and GBM8 expressed roughly similar amounts of N-cadherin mRNA, higher than the level expressed in GBM1, GBM6, GBM7 and GBM9; of these tumours, GBM5 was highly invasive while GBM8 was

**Figure 1** N-cadherin and α-catenin mRNA levels in astrocytomas. The figure shows an autoradiograph of Northern blot analysis of total RNA (20 μg per lane) extracted from glioblastoma multiforme tumours (GBM) classified as WHO grade IV; anaplastic astrocytomas (AGrIII) classified as WHO grade III; low grade astrocytomas (LGA) classified as WHO grade II; a pilocytic astrocytoma (PILO) classified as WHO grade I; and a normal brain sample (NB). In one case (GBM5) two different samples from the same tumour were analysed. Blots were hybridised with cDNA probes for N-cadherin (top) and α-catenin (middle) and with an oligonucleotide probe for 18S rRNA (bottom).

**Table I** Analysis of N-cadherin and α-catenin mRNA levels in gliomas

| *Tumour*[a] | *N-cadherin*[b] | *α-catenin*[b] |
|---|---|---|
| *High-grade gliomas* | | |
| GBM1 | 0.2, 0.2 | 0.2, 0.2 |
| GBM2 | 0.61, 0.58 | 0.35, 0.70 |
| GBM3 | 0.70, 0.32 | 0.54, 0.35 |
| GBM4 | 0.50, 1.05 | 0.48, 0.73 |
| GBM5 | 0.60, 0.57 | 0.46, 0.40 |
| GBM6 | 0.31 | 0.30 |
| GBM7 | 0.26 | 0.24 |
| GBM8 | 0.47 | 0.48 |
| GBM9 | 0.27 | 0.58 |
| AGrIII1 | 0, 0.75 | 0, 0.68 |
| AGrIII2 | 0.3, 0 | 0.33, 0 |
| AGrIII3 | 0 | 0.13 |
| *Low-grade gliomas* | | |
| LGA1 | 0, 0 | 0, 0 |
| LGA2 | 0.19, 0 | 0.14, 0 |
| LGA3 | 0, 0.09 | 0, 0.33 |
| LGA5 | 0.12, 0.21 | 0.12, 0.17 |
| *Other tumours* | | |
| PILO | 0.11, 0.12 | 0.17, 0.30 |
| MG1 | 0 | 0 |
| MG2 | 0 | 0 |
| OLIGO1 | 0 | 0 |
| OLIGO2 | 0 | 0 |
| *Control tissues* | | |
| Normal brain | 0.18 | 0.07 |
| Heart | 0.72, 0.95 | 0.42, 0 |
| Liver | 0, 0.17 | 0, 0 |
| Placenta | 0, 0 | 0, 0 |

[a]RNA was extracted from tumour specimens (see legend to Figure 1), analysed by Northern blot and quantified by use of a phosphorimager, as described in Materials and methods. The table presents data from the blot shown in Figure 1 combined with data from other similar blots. [b]Relative hybridisation intensities were determined by use of a phosphorimager. The values for N-cadherin or α-catenin were corrected for variations in loading by dividing by the values obtained for 18S RNA. For ease of comparison, all N-cadherin and α-catenin values were normalised to the values obtained from tumour GBM1, which were set to 0.2.

minimally invasive. As another example, within grade III, AGIII2 was highly invasive, whereas AGIII3 was very localised; when corrected for differences in loading, these tumours expressed similar levels of N-cadherin mRNA. Finally, although there was significant variation in the level of mRNAs for N-cadherin and α-catenin in different gliomas (Table I), the ratio between the two mRNAs was generally similar (linear regression and correlation analysis yielded values of $r = 0.838$ and $P = 0.000$).

We also compared the expression of mRNA for N-cadherin and the associated protein α-catenin in several established human glioblastoma cell lines. RNA was extracted from cells harvested at different states of confluence analysed by Northern blots. The seven glioblastoma cell lines also expressed elevated levels of N-cadherin and α-catenin mRNA relative to normal brain (Figure 2 and Table II). In contrast to the tumour specimens, however, there was no correlation between the levels of N-cadherin and α-catenin mRNA in the cell lines. In addition, mRNA levels for both molecules varied as a function of cell density, although there was no consistent pattern of density-dependent expression among the various cell lines.

*Expression of N-cadherin protein in gliomas and glioblastoma cell lines*

To assess the levels of N-cadherin protein, tumour samples and glioblastoma cell lines were extracted with non-ionic detergents. Equal volumes of each extract were separated by gel electrophoresis, transferred to nitrocellulose and blotted with antibodies against N-cadherin. Subsequent protein determinations indicated that roughly equal amounts of protein were loaded. As shown in Figure 3, antibodies to N-cadherin detected a band of approximately 120 kDa in extracts of all astrocytomas and in normal brain that co-migrated with a band in the positive control extract from chicken heart. The different astrocytoma tumour samples generally contained similar levels of N-cadherin. Although slightly lower levels of N-cadherin were seen in the GBM11 extract, the pattern of amido black staining suggested that there was some degradation of the proteins in this extract. N-cadherin was also detected in extracts of all glioblastoma cell lines (Figure 4), although the levels varied somewhat. The U-87MG and T98G lines expressed relatively low levels of N-cadherin, while the U-373MG and SNB-19 lines expressed relatively high levels.

**Figure 2** mRNA expression of N-cadherin and α-catenin in glioblastoma cell lines at different cell densities. The levels of mRNA for N-cadherin (top) and α-catenin (middle) isolated from seven glioblastoma cell lines at different cell densities were assessed by Northern blotting as described in the legend to Figure 1. RNA was extracted from cells that were semiconfluent (SC), immediately after the cells became confluent (IC) or 4–5 days after the cells became confluent (C). Normal brain (NB) was used as a positive control.

**Figure 3** Protein expression of N-cadherin in astrocytomas and glioblastoma cell lines. Relative levels of N-cadherin protein in extracts from astrocytomas were determined by immunoblot analysis using a polyclonal antiserum raised against a synthetic peptide from the cytoplasmic domain of N-cadherin (Lagunowich *et al*., 1990) as described in Materials and methods. The lower part of each filter was stained with amido black to verify the loading and transfer of extracts. Abbreviations used are: GBM, glioblastoma multiforme; LGA, low-grade astrocytoma; PILO, pilocytic astrocytoma; NB, normal brain; CH, chicken heart.

**Table II** Analysis of N-cadherin and α-catenin mRNA levels in glioblastoma cell lines

| *Cell line*[a] | *N-cadherin*[b] | *α-catenin*[b] |
|---|---|---|
| T98G(SC) | 0.71 | 0.71 |
| T98G(IC) | 0.40 | 0.97 |
| T98G(C) | 0.36 | 1.90 |
| U-373MG(SC) | 1.59 | 0.91 |
| U-373MG(IC) | 0.83 | 0.53 |
| U-373MG(C) | 1.21 | 1.03 |
| SNB-19(SC) | 0.70 | 0.84 |
| SNB-19(IC) | 1.00 | 1.00 |
| SNB-19(C) | 0.71 | 0.95 |
| Hs683(SC) | 0.79 | 0.85 |
| Hs683(C) | 0.71 | 0.84 |
| U-118MG(SC) | 0.68 | 0.63 |
| U-118MG(C) | 0.83 | 0.92 |
| U-87MG(SC) | 0.38 | 0.84 |
| U-87MG(C) | 0.24 | 0.72 |
| U-138MG(SC) | 0.29 | 0.78 |
| U-138MG(C) | 0.63 | 0.77 |

[a]RNA was harvested from indicated cell lines at different stages of confluency – semiconfluent (SC), immediately after reaching confluency (IC) or 4–5 days after reaching confluency (C) – and analysed by Northern blot. [b]Relative hybridisation intensities were determined by use of a phosphorimager. The values for N-cadherin or α-catenin were corrected for variations in loading by dividing by the values obtained for 18S RNA. For ease of comparison, all N-cadherin and α-catenin values were normalised to the values obtained from SNB-19 (IC), which were set to 1.0.

**Figure 4** Protein expression of N-cadherin in astrocytomas and glioblastoma cell lines. Relative levels of N-cadherin protein in extracts from glioblastoma cell lines were determined by immunoblot analysis using a polyclonal antiserum raised against a synthetic peptide from the cytoplasmic domain of N-cadherin (Lagunowich *et al*., 1990) as described in Materials and methods. The lower part of each filter was stained with amido black to verify the loading and transfer of extracts. Abbreviations used are: NB, normal brain; CH, chicken heart.

Table III Cell aggregation and wound filling assays of glioblastoma cell lines

| *Cell line* | *Cell adhesion*[a] | *Wound filling*[b] |
|---|---|---|
| U-87MG | 40% (7%) | 20 h |
| U-118MG | 65% (4%) | NF |
| U-138MG | 74% (7%) | 60 h |
| U-373MG | 56% (21%) | 56 h |
| T98G | 19% (4%) | 68 h |
| SNB-19 | 39% (9%) | 20 h |
| Hs683 | 39% (16%) | 10 days |

[a]Each cell line was analysed in three aggregation assays, as described in Materials and methods. The table shows the average values with standard deviations in parentheses. [b]Wound filling assays were carried out twice, in triplicate, and the table shows the values that were repeatedly obtained. NF, the wound was not filled completely.

### *Cell aggregation and motility of glioblastoma cell lines*

To test whether cadherin-mediated adhesion regulates cell motility in the glioblastoma cell lines, we measured the aggregation and motility of these cells *in vitro* and correlated the results of these two assays with determinations of N-cadherin expression. For cell aggregation assays, cells were removed from culture dishes under conditions that retain cadherins and were tested for their ability to reassociate. As shown in Table III, the different glioblastoma lines showed significant variation in calcium-dependent adhesion, ranging from 19% (T98G) to more than 50% (U-138MG, U-118MG and U-373MG).

To assess the motility of these cells, we employed an *in vitro* assay that measures the ability of the cells to refill a 'wound' introduced into a confluent monolayer of cells by scraping a path. U-87MG showed the shortest time for wound healing (20 h), while the wound healing times of U-118MG and Hs683 were very long (Table III). The times required for refilling the wound varied substantially among the glioblastoma cell lines and showed no consistent relationship to the level of adhesiveness, as revealed in the cell aggregation assays. In addition, motility in the wound refilling assay did not show a consistent correlation with expression of N-cadherin.

### Discussion

Previous work suggests that the loss of E-cadherin that occurs as tumours progress to less differentiated forms is an important determinant of enhanced invasiveness (Takeichi, 1993). N-cadherin is the predominant cadherin in brain tissues and appears to play a major role in maintaining the multicellular structure of brain (Hatta *et al.*, 1985). We therefore tested whether expression of N-cadherin might regulate the invasive behaviour of astrocytomas. We found that N-cadherin and α-catenin expression increases or remains the same as astrocytomas progress to high-grade glioblastomas. In addition, no correlation was observed between cadherin expression and cell motility in a series of glioblastoma cell lines. As discussed below, these results indicate that the relationship between N-cadherin expression and invasion is quite different from that of E-cadherin.

To analyse the relationship between cell adhesion and invasiveness of astrocytomas, we first surveyed the expression of several cell adhesion molecules, including N-cadherin, N-CAM, L1, E-cadherin and P-cadherin, in tumour samples. Results from this initial survey (data not shown) indicated that only N-cadherin and N-CAM, which contributes to cell adhesion to a lesser extent than N-cadherin (Duband *et al.*, 1987), showed appreciable expression. The remainder of our studies focused, therefore, on N-cadherin and the associated protein, α-catenin.

In tumours, higher levels of mRNA for N-cadherin were found in anaplastic astrocytomas (AA) and glioblastomas (GBM) than in low-grade astrocytomas or normal brain. This observation was consistent, in that 8/11 AAs or GBMs showed increased levels, although there was some variation in the magnitude of this increase. If increased invasive capacity resulted in part from loss of N-cadherin, then higher grade gliomas might have been expected to show reduced levels of N-cadherin expression, as is the case for E-cadherin in adenocarcinomas (see Takeichi, 1993). These findings thus suggested that loss of N-cadherin-mediated adhesion was not a key determinant in the enhanced invasion of higher grade tumours. One limitation to this analysis was that, although higher grade tumours are generally more invasive, this is not invariably the case. We therefore compared the expression of N-cadherin among tumours within the same grade that were judged, by examination of MR scans, to be highly or minimally invasive. This analysis showed that there is no consistent relationship between invasion and N-cadherin expression, confirming the conclusion that alterations in N-cadherin levels do not appear to contribute to acquisition of invasive capacity.

It was striking that, in all of the tumours examined, increases in N-cadherin mRNA were accompanied by approximately proportional increases in the levels of α-catenin mRNA. The α-catenin probe used recognises both α-E-catenin and α-N-catenin, but only α-N-catenin is expressed in normal brain (Hirano *et al.*, 1992). We presume therefore that the elevated levels of α-catenin mRNA detected in tumours correspond to α-N-catenin. If the correlation between increased N-cadherin mRNA and α-catenin mRNA is not simply fortuitous, this finding may suggest that expression of the two genes is coordinately controlled. In contrast, α-catenin expression is greatly reduced in some epithelial tumours that retain high levels of E-cadherin (Shimoyama *et al.*, 1992; Morton *et al.*, 1993; Kadowaki *et al.*, 1994), apparently contributing to the invasive ability of the tumour cells.

In contrast to the increases in mRNA for N-cadherin, immunoblot analysis suggested that normal brain, low-grade astrocytomas and high-grade astrocytomas all contained roughly similar levels of intact N-cadherin protein. Although this apparent discrepancy could arise in several ways, one intriguing possibility is that the N-cadherin protein produced in the high-grade tumours is not completely destroyed, but, by analogy with the situation known to occur during normal retinal development (Paradies and Grunwald, 1993), is cleaved to produce a soluble fragment that might retain some biological activity. This possibility is consistent with our findings, but could not be directly tested, because the antibody used to detect N-cadherin reacts with the carboxyl-terminal portion of the molecule, and thus would not detect the soluble fragment.

Chen and Öbrink (1991) have observed that cell contact mediated by E-cadherin inhibits cell motility and have suggested that this may be the means by which cadherins inhibit invasion. We therefore evaluated the motility, calcium-dependent adhesion and N-cadherin expression of several human glioblastoma cell lines, to analyse further whether there is any relationship between cadherin-mediated adhesion and invasion of glioblastoma cells. As in the glioblastoma tumours, all seven lines showed higher levels of N-cadherin mRNA expression than found in normal brain or low-grade astrocytomas. There were significant variations in calcium-dependent adhesion among the different cell lines and, given that other neural cadherins might be variably expressed by these lines, it was perhaps not surprising that the variations in adhesion did not appear to reflect differences in the levels of N-cadherin expression. The lines also showed significant variations in motility, as assessed by their ability to refill 'wounds' made in a confluent monolayer of cultured cells (the same assay used by Chen and Öbrink). In contrast to the relationship between E-cadherin mediated adhesion and motility (Chen and Öbrink, 1991), no consistent relationship was seen in the glioblastoma cell lines between cadherin expression and motility, although such a correlation could have been obscured by variations in intrinsic levels of motility.

The experiments described here were motivated by studies of the relationship between E-cadherin expression and invasion in carcinoma cells (Takeichi, 1993). The results of our studies strongly suggest that changes in expression of N-cadherin that occur during tumour progression differ from those seen with E-cadherin and, further, that N-cadherin does not appear to inhibit cell motility as E-cadherin does (Chen and Öbrink, 1991). These conclusions are further supported by our recent studies on the effects of E-cadherin and N-cadherin on the motility and invasion of an astrocyte-like cell line, WC5 (Paradies *et al.*, 1995). In those experiments, E-cadherin expression reduced motility and invasion of WC5 cells, whereas expression of N-cadherin had little effect on the rate of invasion.

In conclusion, these studies show that the expression of N-cadherin mRNA and protein remain at equal or higher levels in high-grade astrocytomas relative to low-grade astrocytoma or normal brain. Together with the finding that glioblastoma cell lines show no consistent relationship between N-cadherin expression and cell motility, the results imply that expression of N-cadherin does not regulate invasion of high-grade astrocytomas.

**Acknowledgements**
We thank Jerry Grunwald (Thomas Jefferson University, Philadelphia, PA, USA) for generously providing a polyclonal antibody against N-cadherin. This work was supported by NIH Grant P20-NS31145. NS was supported by a fellowship from the International Medical Center (Tokyo, Japan) and NEP by a Dale DiVenti Fellowship from the American Brain Tumor Association. HC was supported, in part, by NCI Training Grant CA59268.

### References

ALBELDA SM AND BUCK CA. (1990). Integrins and other cell adhesion molecules. *FASEB J.*, **4**, 2868–2880.

BEHRENS J, MAREEL MM, VAN ROY FM AND BIRCHMEIER W. (1989). Dissecting tumor cell invasion: epithelial cells acquire invasive properties after the loss of uvomorulin-mediated cell–cell adhesion. *J. Cell Biol.*, **108**, 2435–2447.

BRACKENBURY R, THIERY JP, RUTISHAUSER U AND EDELMAN GM. (1977). Adhesion among neural cells of the chick embryo. *J. Biol. Chem.*, **252**, 6835–6840.

BRACKENBURY R, RUTISHAUSER U AND EDELMAN GM. (1981). Distinct calcium-independent and calcium-dependent adhesion systems of chicken embryo cells. *Proc. Natl Acad. Sci. USA*, **78**, 387–391.

BURGER PC, HEINZ ER, SHIBATA T AND KLEIHUES P. (1988). Topographic anatomy and CT correlations in the untreated glioblastoma multiforme. *J. Neurosurg.*, **68**, 698–704.

CHEN W AND ÖBRINK B. (1991). Cell-cell contacts mediated by E-cadherin (uvomorulin) restrict invasive behavior of L-cells. *J. Cell Biol.*, **114**, 319–327.

CHOMCZYNSKI P AND SACCHI N. (1987). Single-step method of RNA isolation by acid guanidinium thiocyanate–phenol–chloroform extraction. *Anal. Biochem.*, **162**, 156–159.

CHURCH GM AND GILBERT W. (1984). Genomic sequencing. *Proc. Natl Acad. Sci. USA*, **81**, 1991–1995.

COMAN DR. (1944). Decreased mutual adhesiveness, a property of cells from squamous cell carcinomas. *Cancer Res.*, **4**, 625–629.

COMAN DR. (1947). Mechanism of the invasiveness of cancer. *Science*, **105**, 347–348.

CUNNINGHAM BA, EDELMAN GM AND THIERY J-P (eds). (1990). *Morphoregulatory Molecules*. John Wiley: New York.

DOHERTY P, ASHTON SV, MOORE SE AND WALSH FS. (1991). Morphoregulatory activities of N-CAM and N-cadherin can be accounted for by G protein-dependent activation of L- and N-type neuronal $Ca^{2+}$ channels. *Cell*, **67**, 21–33.

DUBAND JL, DUFOUR S, HATTA K, TAKEICHI M, EDELMAN GM AND THIERY JP. (1987). Adhesion molecules during somitogenesis in the avian embryo. *J. Cell Biol.*, **104**, 1361–1374.

FRIXEN UH, BEHRENS J, SACHS M, EBERLE G, VOSS B, WARDA A, LOCHNER D AND BIRCHMEIER W. (1991). E-cadherin-mediated cell–cell adhesion prevents invasiveness of human carcinoma cells. *J. Cell Biol.*, **113**, 173–185.

GROSS JL, BEHRENS DL, MULLINS DE, KORNBLITH PL AND DEXTER DL. (1988). Plasminogen activator and inhibitor activity in human glioma cells and modulation by sodium butyrate. *Cancer Res.*, **48**, 291–296.

HATTA K AND TAKEICHI M. (1986). Expression of N-cadherin adhesion molecules associated with early morphogenetic events in chick development. *Nature*, **320**, 447–449.

HATTA K, OKADA TS AND TAKEICHI M. (1985). A monoclonal antibody disrupting calcium-dependent cell–cell adhesion of brain tissues: possible role of its target antigen in animal pattern formation. *Proc. Natl Acad. Sci. USA*, **82**, 2789–2793.

HATTA K, TAKAGI S, FUJISAWA H AND TAKEICHI M. (1987). Spatial and temporal expression pattern of N-cadherin cell adhesion molecule correlated with morphogenetic processes of chicken embryos. *Dev. Biol.*, **120**, 215–227.

HIRANO S, KIMOTO N, SHIMOYAMA Y, HIROHASHI S AND TAKEICHI M. (1992). Identification of a neural α-catenin as a key regulator of cadherin function and multicellular organization. *Cell*, **70**, 293–301.

INUZUKA H, MIYATANI S AND TAKEICHI M. (1991). R-cadherin: a novel $Ca^{2+}$-dependent cell-cell adhesion molecule expressed in the retina. *Neuron*, **7**, 69–79.

KADOWAKI T, SHIOZAKI H, INOUE M, TAMURA S, OKA H, DOKI Y, IHARA K, MATSUI S, IWAZAWA T, NAGAFUCHI A, TSUKITA S AND MORI T. (1994). E-cadherin and α-catenin expression in human esophageal cancer. *Cancer Res.*, **54**, 291–296.

KELLY PH, DAUMAS-DUPORT C, KISPERT DB, KALL BA, SCHEITHAUER BW AND ILLIG JJ. (1987). Imaging-based stereotaxic serial biopsies in untreated intracranial glial neoplasms. *J. Neurosurg.*, **66**, 865–874.

LAEMMLI UK. (1970). Cleavage of structural proteins during the assembly of the head of bacteriophage T4. *Nature*, **227**, 680–685.

LAGUNOWICH LA, DONOSO LA AND GRUNWALD GB. (1990). Identification of mammalian and invertebrate analogues of the avian calcium-dependent cell adhesion protein N-cadherin with synthetic peptide directed antibodies against a conserved cytoplasmic domain. *Biochem. Biophys. Res. Commun.*, **172**, 313–320.

LIOTTA LA, STEEG PS AND STETLER-STEVENSON WG. (1991). Cancer metastasis and angiogenesis: an imbalance of positive and negative regulation. *Cell*, **64**, 327–336.

McCALLUM FS AND MADEN BEH. (1985). Human 18S ribosomal RNA sequence inferred from DNA sequence. *Biochem. J.*, **232**, 725–733.

MAYER B, JOHNSON JP, LEITL F, JAUCH KW, HEISS MM, SCHILDBERG FW, BIRCHMEIER W AND FUNKE I. (1993). E-cadherin expression in primary and metastatic gastric cancer: down-regulation correlates with cellular differentiation and glandular disintegration. *Cancer Res.*, **53**, 1690–1695.

MORTON RA, EWING CM, NAGAFUCHI A, TSUKITA S AND ISAACS WB. (1993). Reduction of E-cadherin levels and deletion of the α-catenin gene in human prostate cancer cells. *Cancer Res.*, **53**, 3585–3590.

NAGAFUCHI A AND TAKEICHI M. (1988). Cell binding function of E-cadherin is regulated by the cytoplasmic domain. *EMBO J.*, **7**, 3679–3684.

NAGAFUCHI A, SHIRAYOSHI Y, OKAZAKI K, YASUDA K AND TAKEICHI M. (1987). Transformation of cell adhesion properties by exogenously introduced E-cadherin cDNA. *Nature*, **329**, 341–343.

NAPOLITANO EW, VENSTROM K, WHEELER EF AND REICHARDT LF. (1991). Molecular cloning and characterization of B-cadherin, a novel chick cadherin. *J. Cell Biol.*, **113**, 893–905.

NOSE A, TSUJI K AND TAKEICHI M. (1990). Localization of specificity determining sites in cadherin cell adhesion molecules. *Cell*, **61**, 147–155.

ODA T, KANAI Y, SHIMOYAMA Y, NAGAFUCHI A, TSUKITA S AND HIROHASHI S. (1993). Cloning of the human α-catenin cDNA and its aberrant mRNA in a human cancer cell line. *Biochem. Biophys. Res. Commun.*, **193**, 897–904.

OWENS RB, SMITH HS, NELSON-REES WA AND SPRINGER EL. (1976). Brief communication: epithelial cell cultures form normal and cancerous human tissues. *J. Natl Cancer Inst.*, **56**, 843–849.

OZAWA M AND KEMLER R. (1992). Molecular organization of the uvomorulin–catenin complex. *J. Cell Biol.*, **116**, 989–996.

OZAWA M, BARIBAULT H AND KEMLER R. (1989). The cytoplasmic domain of the cell adhesion molecule uvomorulin associates with three independent proteins structurally related in different species. *EMBO J.*, **8**, 1711–1717.

OZAWA M. RINGWALD M AND KEMLER R. (1990). Uvomorulin-catenin complex formation is regulated by a specific domain in the cytoplasmic region of the cell adhesion molecule. *Proc. Natl Acad. Sci. USA*, **87**, 4246-4250.

PARADIES NE AND GRUNWALD GB. (1993). Purification and characterization of NCAD90, a soluble endogenous form of N-cadherin, which is generated by proteolysis during retinal development and retains adhesive and neurite-promoting function. *J. Neurosci. Res.*, **36**, 33-45.

PARADIES NE, KLOPFENSTEIN KJ AND BRACKENBURY R. (1995). E-cadherin, but not N-cadherin, inhibits motility and reduces invasion of RSV-transformed WC5 rat cerebellar cells. *J. Cell Biol.*, submitted for publication.

PONTEN J AND MACINTYRE EH. (1968). Long term culture of normal and neoplastic human glia. *Acta Pathol. Microbiol. Scand.*, **74**, 465-486.

RANSCHT B AND DOURS-ZIMMERMAN MT. (1991). T-cadherin, a novel cadherin cell adhesion molecule in the nervous system lacks the conserved cytoplasmic region. *Neuron*, **7**, 391-402.

RASBRIDGE SA, GILLETT CE, SAMPSON SA, WALSH FS AND MILLIS RR. (1993). Epithelial (E-) and placental (P-) cadherin cell adhesion molecule expression in breast carcinoma. *J. Pathol.*, **169**, 245-250.

REID RA AND HEMPERLY JJ. (1990). Human N-cadherin: nucleotide and deduced amino acid sequence. *Nucleic Acids Res.*, **18**, 5896.

SCHIPPER JH, FRIXEN UH, BEHRENS J, UNGER A, JAHNKE K AND BIRCHMEIER W. (1991). E-cadherin expression in squamous cell carcinomas of head and neck: inverse correlation with tumor dedifferentiation and lymph node metastasis. *Cancer Res.*, **51**, 6328-6337.

SELDEN RF. (1992). Analysis of RNA by Northern hybridization. In *Current Protocols in Molecular Biology*, Ausubel FM, Brent R, Kingston RE, Moore DD, Seidman JG, Smith JA and Struhl K. (eds) pp. 4.9.1-4.9.8. Greene Publishing Associates and Wiley-Interscience: Boston.

SHIOZAKI H, TAHARA H, OKA H, MIYATA M, KOBAYASHI K, TAMURA S, IHARA K, DOKI Y, HIRANO S, TAKEICHI M AND MORI T. (1991). Expression of E-cadherin molecules in human cancers. *Am. J. Pathol.*, **139**, 17-23.

SHIMOYAMA Y, NAGAFUCHI A, FUJITA S, GOTOH M, TAKEICHI M, TSUKITA S AND HIROHASHI S. (1992). Cadherin dysfunction in a human cancer cell line: possible involvement of loss of α-catenin expression in reduced cell-cell adhesiveness. *Cancer Res.*, **52**, 5770-5774.

SHIRAYOSHI Y, OKADA TS AND TAKEICHI M. (1983). The calcium-dependent cell-cell adhesion system regulates inner cell mass formation and cell surface polarization in early mouse development. *Cell*, **35**, 631-638.

SOMMERS CL, THOMPSON EW, TORRI JA, KEMLER R, GELMANN EP AND BYERS SW. (1991). Cell adhesion molecule uvomorulin expression in human breast cancer cell lines: relationship to morphology and invasive capacities. *Cell Growth Different.*, **2**, 365-372.

STEIN GH. (1979). T98G: an anchorage-independent human tumor cell line that exhibits stationary phase G1 arrest in vitro. *J. Cell. Physiol.*, **99**, 43-54.

SUZUKI S, SANO K AND TANIHARA H. (1991). Diversity of the cadherin family: evidence for eight new cadherins in nervous tissue. *Cell Regulat.*, **2**, 261-270.

TAKEICHI M. (1988). The cadherins: cell-cell adhesion molecules controlling animal morphogenesis. *Development*, **102**, 639-655.

TAKEICHI M. (1993). Cadherins in cancer: implications for invasion and metastasis. *Curr. Opin. Cell Biol.*, **5**, 806-811.

TAKEICHI M, ATSUMI T, YOSHIDA C, UNO K AND OKADA TS. (1981). Selective adhesion of embryonal carcinoma cells and differentiated cells by $Ca^{2+}$-dependent sites. *Dev. Biol.*, **87**, 340-350.

TOMASELLI KJ, NEUGEBAUER KM, BIXBY JL, LILIEN J AND REICHARDT LF. (1988). N-cadherin and integrins: two-receptor systems that mediate neuronal process outgrowth on astrocytes. *Neuron*, **1**, 33-43.

VLEMINCKX K, VAKAET Jr L, MAREEL M, FIERS W AND ROY FV. (1991). Genetic manipulation of E-cadherin expression by epithelial tumor cells reveals an invasion suppressor role. *Cell*, **66**, 107-119.

WALSH FS, BARTON CH, PUTT W, MOORE SE, KELSELL D, SPURR N AND GOODFELLOW PN. (1990). N-cadherin gene maps to human chromosome 18 and is not linked to the E-cadherin gene. *J. Neurochem.*, **55**, 805-812.